# Mesenchymal stem cell‐derived exosomes improve neurogenesis and cognitive function of mice with methamphetamine addiction: A novel treatment approach

**DOI:** 10.1111/cns.14719

**Published:** 2024-05-23

**Authors:** Solmaz Fallahi, Hamid Soltani Zangbar, Fereshteh Farajdokht, Reza Rahbarghazi, Fariba Ghiasi, Gisou Mohaddes

**Affiliations:** ^1^ Drug Applied Research Center Tabriz University of Medical Sciences Tabriz Iran; ^2^ Department of Physiology Tabriz University of Medical Sciences Tabriz Iran; ^3^ Department of Neuroscience and Cognition, Faculty of Advanced Medical Sciences Tabriz University of Medical Sciences Tabriz Iran; ^4^ Neurosciences Research Center Tabriz University of Medical Sciences Tabriz Iran; ^5^ Department of Applied Cell Sciences, Faculty of Advanced Medical Sciences Tabriz University of Medical Sciences Tabriz Iran; ^6^ Department of Biomedical Education California Health Sciences University, College of Osteopathic Medicine Clovis California USA

**Keywords:** addiction, bone marrow mesenchymal stem cells, cognition, exosome, methamphetamine, neurogenesis, neuroinflammation

## Abstract

**Background:**

Methamphetamine (METH) is a psychostimulant substance with highly addictive and neurotoxic effects, but no ideal treatment option exists to improve METH‐induced neurocognitive deficits. Recently, mesenchymal stem cells (MSCs)‐derived exosomes have raised many hopes for treating neurodegenerative sequela of brain disorders. This study aimed to determine the therapeutic potential of MSCs‐derived exosomes on cognitive function and neurogenesis of METH‐addicted rodents.

**Methods:**

Male BALB/c mice were subjected to chronic METH addiction, followed by intravenous administration of bone marrow MSCs‐derived exosomes. Then, the spatial memory and recognition memory of animals were assessed by the Barnes maze and the novel object recognition test (NORT). The neurogenesis‐related factors, including NeuN and DCX, and the expression of Iba‐1, a microglial activation marker, were assessed in the hippocampus by immunofluorescence staining. Also, the expression of inflammatory cytokines, including TNF‐α and NF‐κB, were evaluated by western blotting.

**Results:**

The results showed that BMSCs‐exosomes improved the time spent in the target quadrant and correct‐to‐wrong relative time in the Barnes maze. Also, NORT's discrimination index (DI) and recognition index (RI) were improved following exosome therapy. Additionally, exosome therapy significantly increased the expression of NeuN and DCX in the hippocampus while decreasing the expression of inflammatory cytokines, including TNF‐α and NF‐κB. Besides, BMSC‐exosomes down‐regulated the expression of Iba‐1.

**Conclusion:**

Our findings indicate that BMSC‐exosomes mitigated METH‐caused cognitive dysfunction by improving neurogenesis and inhibiting neuroinflammation in the hippocampus.

## Graphical abstract

Following methamphetamine addiction, the level of inflammation in the hippocampus of mice increases, and neurogenesis decreases, which leads to a decline in cognitive function. Exosome therapy changes the process by reducing the expression of inflammatory factors (TNFα, NFkβ, Iba1) and increasing the neurogenesis‐related factors (DCX, NeuN), improving cognitive function.

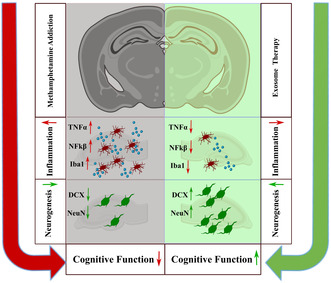

## 1 INTRODUCTION

Methamphetamine (METH) is a widely used psychostimulant substance that readily crosses the blood–brain barrier (BBB) and enters the brain.^1^ Adverse consequences resulting from its abuse have emerged as a worldwide concern,^2^ as it impairs the functionality and cognitive processes of the central nervous system (CNS).^2^, ^3^ METH disrupts the normal neuronal activity in the brain by enhancing the release of neurotransmitters such as dopamine, serotonin, and glutamate.^4^ Prolonged methamphetamine abuse is associated with neuronal loss in various brain regions, including the substantia nigra pars compacta and locus coeruleus, resulting in the deterioration of different cognitive domains, including memory and decision‐making.^5^, ^6^, ^7^ Kami et al. reported that repetitive METH treatment impairs working memory through suppression of the ERK1/2 pathway in the hippocampus.^8^ Furthermore, the cognitive decline induced by METH may be associated with the activation of inflammatory responses and the upregulation of pro‐inflammatory cytokines in the hippocampus.^9^ A postmortem study reported that glial fibrillary acidic protein (GFAP) level, a marker of astroglial injury, and ionized calcium‐binding adaptor molecule 1 (Iba‐1) level, a microglial marker, are increased in the hippocampal CA1 region of METH‐addicted subjects.^10^ Besides, METH decreases the proliferation, survival, and neurogenesis of neural stem cells in the dentate gyrus (DG) of the hippocampus.^11^ METH exposure during pregnancy or lactation dramatically reduces the expression of doublecortin (DCX)‐positive cells, as a marker of neurogenesis, in the hippocampal DG area of the offspring, impairing their long‐term and spatial memory later in life.^12^

Several pharmacological interventions and therapeutic strategies have been proposed to mitigate the detrimental impact of METH on the CNS, though the majority of these approaches encounter difficulties.^13^, ^14^ In recent years, the application of mesenchymal stem cells (MSCs) has emerged as a prominent strategy for addressing neurological disorders in experimental studies.^15^ MSCs substantially decrease the production of pro‐inflammatory cytokines, such as TNF‐α, IL‐6, and IL‐1β, while boosting the levels of M2 macrophages and anti‐inflammatory cytokines, such as IL‐10, in the injured brain tissue.^16^ These cells also activate the BDNF/TrkB/CREB signaling pathway, leading to elevated levels of DCX, PSD‐95, and synaptophysin, thereby proving the occurrence of neurogenesis and synaptogenesis.^17^ MSC‐derived exosomes are small lipid vesicles (30 to 150 nm in diameter) that are loaded with numerous biological components, including mRNA, miRNA, proteins, cytokines, lipids, and other molecules.^18^ Additionally, exosomes are less immunogenic than their progenitor cells, presenting no risk of tumorigenesis, and are easier to produce and store.^19^ Exosomes mimic the therapeutic abilities of MSCs and efficiently enhance neurogenesis while reducing cognitive deficits caused by neurodegenerative disorders.^20^, ^21^ Exosomes suppress neuroinflammation by reducing the levels of inflammatory mediators such as IL‐1β, TNF‐α, and IL‐6, and inhibiting the NFk‐β/ERK/JNK signaling pathways in glial cells.^22^ Furthermore, exosomes have the ability to traverse the BBB and stimulate the growth of new neurons in the hippocampus. They also offer protection to the nervous system against oxidative damage in several neurological diseases.^23^, ^24^ Intranasal (IN) administration of human MSC‐derived extracellular vesicles in traumatic brain injury (TBI) mice increases neurogenesis in the sub‐ventricular zone (SVZ) by increasing cell proliferation and enhancing the expression of DCX and NeuN.^25^ Pourhadi et al. also demonstrated that intranasal exosomes reduce amyloid‐beta deposits and increase hippocampus pre‐ and post‐synaptic protein expression to improve spatial memory in Alzheimer's disease model.^26^

Based on the above, we aimed to investigate the impact of intravenous exosome delivery on cognitive function and neurogenesis markers in the DG and CA1 region of the hippocampus in METH‐treated mice.

## 2 MATERIALS AND METHODS

### 2.1 Animals and drugs

**FIGURE 1:**
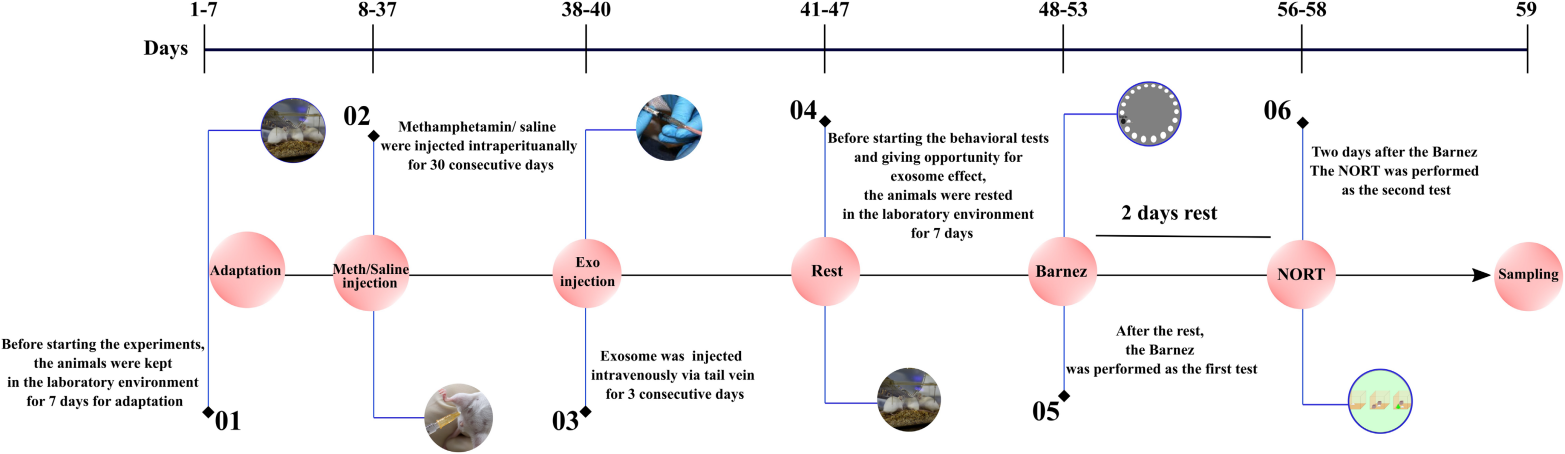
Timeline of the study. Exo, exosomes; Meth, methamphetamine; NORT, novel object recognition test.

A total of 40 adult male BALB/c mice, aged 6 weeks and weighing 20–25 g, were acquired from the Experimental Animal Center of Tabriz University of Medical Sciences (Tabriz, Iran). The animals were allocated into four groups (Table 1) and housed in standard cages with an unrestricted supply of food and water. The ambient temperature in the room was set at a constant 23 ± 1°C, and the lighting conditions followed a 12‐h cycle with lights turned off at 7 p.m. The Counter Narcotics Headquarters provided METH. The METH was dissolved in distilled water and administered intraperitoneally at a dosage of 5 mg/kg. The timeline of the study is presented in Figure 1.

**TABLE 1:** The study groups and related treatments.

Groups	Treatment drug	Dosage and injection method
Saline	Saline	IP injection, 1 mL/kg/day for 30 days
Meth‐Saline	Methamphetamine	IP injection, 5 mg/kg/day for 30 days
Saline‐Exo	Saline	IP injection, 1 mL/kg/day for 30 days
	Exosome	IV injection, 30 μg/day for 3 days
Meth‐Exo	Methamphetamine	IP injection, 5 mg/kg/daily for 30 days
	Exosome	IV injection, 30 μg/day for 3 days

Abbreviations: Exo, exosome; IP, intraperitoneal; IV, intravenous; Meth, methamphetamine.

### 2.2 Behavioral assessments

Following the administration of exosome treatment for three consecutive days, behavioral assessments were initiated. Initially, the Barnes maze was conducted for 6 days to assess the spatial reference memory. Following 2 days of rest, the animals underwent the novel object recognition test (NORT) to assess their recognition memory.^27^ The tests were captured by a camera positioned on the top of the table and processed using the video tracking program EthoVision™ (Noldus, The Netherlands). Following the completion of the behavioral assessments, tissue sampling, and molecular analysis were conducted.

#### 2.2.1 Barnes maze

The Barnes maze (Figure 4A) is a circular table with 20 holes, where a dark escape container is positioned below the target hole. To aid the animal in identifying its orientation, visual cues were strategically positioned around the maze. On day one, during the habituation phase, the mouse was placed in the center of the table and then guided toward the target box. After the mouse entered the hole, the opening of the hole was sealed, permitting the mouse to remain inside the box for 60 s. During the acquisition phase (from day 2 to day 5), the mouse learned to locate the target box. This was achieved by performing four trials per block for four consecutive days. Each trial lasted for 3 min and there was a 2‐min interval between each trial. The probe test was conducted on the sixth day, during which the target box was removed and each animal was given 3 min to freely move around the maze. After each test, the surface of the table was cleaned using a 70% alcohol solution to prevent the mice from detecting any residual odor signs. The assessment indices in this test include the escape latency time across the acquisition days, time spent in the target quadrant, and correct to wrong relative time in the probe test.^28^

#### 2.2.2 Novel object recognition test (NORT)

The NORT (Figure 5A) was performed to assess recognition memory.^27^ During the first day, the mice were habituated to the chamber for 5 min (habituation phase). Multiple behaviors such as rearing (the frequency of standing on the hind limbs), self‐grooming (rapid cleaning of face and body by forelegs), and the time spent in the center were assessed to determine the anxiety level of the animals. On the second day, two identical objects were positioned in the right and left corners of the chamber, and the animal was allowed to investigate the chamber for 5 min (sample phase). On the following day (24 h later), one of the two familiar objects was substituted with a novel object (selection phase), and the mouse was allowed to explore the objects for 5 min. The recognition memory of each mouse was evaluated by measuring the duration of object exploration and the ability to differentiate the novel object from the previously encountered object.

### 2.3 Isolation and culture of bone marrow mesenchymal stem cells (BMSCs)

BMSCs were obtained from the femur bones of male BALB/c mice (*n* = 5, aged 6–8 weeks, and weighing 18–20 g), according to a standard protocol.^29^ In brief, mice were euthanized, and the bones were promptly harvested and then soaked in 75% alcohol for 15 min, then transferred to the laminar hood. The epiphyses on both sides of the bones were cut using sterile scissors. The bone marrow was extracted using a sterile syringe and transferred into a T75 flask containing low glucose DMEM (Dulbecco's Modified Eagle's media) culture media (Gibco™) supplemented with 15% fetal bovine serum (FBS) (Gibco™) and 1% penicillin–streptomycin (Gibco™). Cells were cultured at 37°C in a 5% CO_2_ incubator environment. The cell culture medium was replaced every 3 days for 15–21 days. Passage was carried out when the cells reached an estimated confluency of 80%, and exosomes were extracted from the cultured stem cells at passages 3 to 5.

#### 2.3.1 Flow cytometry to identify BMSCs

BMSCs were identified by flow cytometry.^30^ For this purpose, the cells were separated from the bottom of the dish using trypsin in the third passage, then rinsed with PBS and permeabilized using a 1% Triton solution. Following multiple washes with PBS, the cells were subjected to the primary antibody under dark conditions at room temperature for 1 h. Next, the cells were incubated with a secondary antibody labeled with fluorescent substances for an hour at room temperature in a dark environment. Following PBS washing, the cells underwent centrifugation, and a fixing buffer containing 1% paraformaldehyde was applied. To determine the proportion of cells that express specific markers for BMSCs,^31^ flow cytometry analysis using fluorescein isothiocyanate (FITC) was performed on CD34 (ab198395), CD45 (ab10558), CD73 (ab288154), and CD90 (ab225). The analysis was conducted using the BD FACSCalibur Flow Cytometer apparatus (USA) and FlowJo software version 10.5.3.

#### 2.3.2 Isolation of BMSCs‐derived exosomes

The exosomes were isolated using the EXOCIB kit (Cibbiotech, Tehran, Iran) according to the manufacturer's protocol. Once the cells in the flask reached a confluency of 90%, the medium containing cells was substituted with an FBS‐free medium and collected after 48 h. Following the instructions, the FBS‐free media were subjected to filtration using a 0.22 μm filter to eliminate any debris and non‐viable cells. Following the centrifugation of the supernatants at 3000 *g* for 20 min, the remaining debris was removed, and reagent A was introduced to the supernatants. The mixture was kept cold at a temperature of 4°C for the duration of one night and subsequently subjected to centrifugation at a force of 3000 *g* for 40 min. The exosome pellets were resuspended using reagent B and stored at a temperature of −70°C.

The characteristics of the isolated exosomes from BMSCs were assessed using western blot techniques^32^ to identify the presence of CD9 (Santa Cruz, sc‐13,118, 1:1000), CD81 (Santa Cruz, sc‐166,029, 1:1000), CD63 (Santa Cruz, sc‐5275, 1:500), and TSG101 (Santa Cruz, sc‐7964, 1:1000). The morphology of exosomes was evaluated using TEM and SEM electron microscopes. The size and distribution of exosomes were determined using dynamic light scattering (DLS) with the Nano ZS instrument from Malvern Instruments, located in Worcester, Worcestershire, UK. The protein levels of exosomes were measured using the PROTOCIB kit (Cibbiotech, Tehran, Iran). The exosomes were labeled with red Dil (Invitrogen™) prior to injection and administered intravenously through the tail vein for three consecutive days. Each injection dose consisted of 30 μg of protein in a volume of treated 100 μL PBS (Table 1).

### 2.4 Molecular and histological evaluations

Following the behavioral tests, the animals were euthanized under deep anesthesia using an intraperitoneal administration of ketamine/xylazine (100/10 mg/kg). Following the removal of the brain, hippocampus tissue was extracted and stored at 70°C for molecular analysis in half of the animals in each group. The remaining half of the animals underwent transcardial perfusion with a 4% paraformaldehyde solution to fix the brain for histological analysis.

#### 2.4.1 Western blotting

The expression of TNF‐α and NF‐ᴋB in the hippocampus was assessed using Western blotting, following a previously established procedure.^33^ The hippocampal tissue was homogenized and sonicated to western blotting in radioimmunoprecipitation assay (RIPA) buffer and then centrifuged. The protein content was determined using the PROTOCIB kit (Cibbiotech, Tehran, Iran) after collecting the supernatant. The protein samples underwent separation on a sodium dodecyl sulfate‐polyacrylamide electrophoresis gel (SDS–PAGE, 12.5%) and were subsequently transferred to PVDF membranes. The membranes were blocked for 1 h at ambient temperature using a 5% solution of low‐fat milk in TBS buffer. Subsequently, the samples were subjected to overnight incubation with primary antibodies targeting NFᴋβ (ab16502, 1:1000) and TNF‐α (ab6671, 1:1000) at a temperature of 4°C. Following the washing step using TBS buffer, the membranes were exposed to a secondary antibody containing horseradish peroxidase for 1.5 h at room temperature. The blots were detected using a chemiluminescence agent, and the bands were analyzed using Image‐J analysis software.

#### 2.4.2 Immunofluorescence staining

The immunofluorescence method was used to assess the expression of DCX, NeuN, and Iba‐1 markers in the DG and CA regions of the hippocampus using a standard procedure.^34^ The brain samples were dehydrated using ethyl alcohol and purified using glycol. Subsequently, the samples were embedded in paraffin, sliced into coronal slices measuring 5 μm, and mounted on slides. Following the washing procedure with a PBS solution, the samples were immersed in a Triton X‐100 solution and PBS at room temperature for an hour, and then put in a blocking solution for a further hour. Following the washing step in PBS buffer, the samples were incubated with DCX (ab167400, Abcam, USA), NeuN (ab104224, Abcam, USA), and Iba1 (E‐AB‐10382) primary antibodies overnight at 4°C. After washing, the slides were exposed to secondary biotinylated antibodies containing peroxidase‐bound streptavidin for an hour. We utilized DAPI staining (Sigma‐Aldrich) to specifically identify the nuclear DNA. Following a 5‐min wash in PBS, the samples were examined using a fluorescence microscope (Zeiss, Axiophot, Germany).

### 2.5 Statistical analysis

The statistical analyses were conducted using GraphPad Prism (version 9.0). The results are shown as the mean ± standard error of the mean (SEM). The data were tested with the Kolmogorov–Smirnov test to check the normality of the data. A two‐way ANOVA followed by a Tukey post hoc test was used to reveal significant differences across different groups in the escape latency of the Barnes maze. The results of the NORT were analyzed by an independent *t*‐test to identify significant differences in the exploration time of each object in each phase. A one‐way ANOVA followed by Tukey post hoc test was used to look at the data from western blotting, immunofluorescence staining, the probe test of the Barnes maze, and the discriminating index (DI) and recognition index (RI) of the NORT. A *p*‐value of <0.05 indicated statistical significance.

## 3 RESULTS

### 3.1 Identification of the isolated BMSCs and exosomes

**FIGURE 2:**
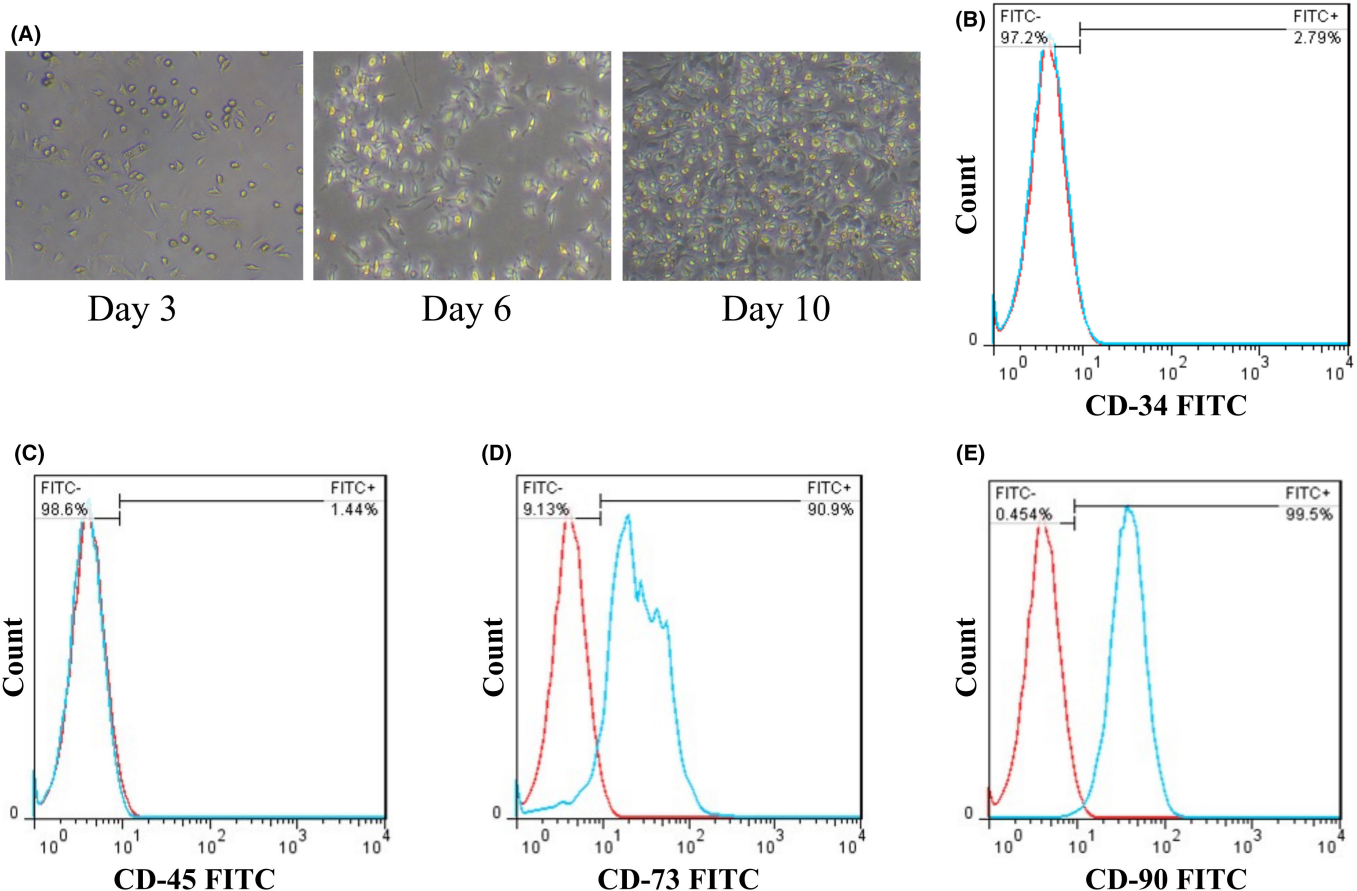
Identification of BMSCs. (A) Spindle‐shaped morphology of BMSCs verified by light microscopic image. (B, C) FITC examination confirms that the third passage cells do not express CD34 and CD45. (D, E) FITC examination confirms that the third passage cells express CD73 and CD90.

BMSCs, after growth and proliferation, were detected with a spindle‐shaped morphology (Figure 2A). Also, the FITC examination confirms that the third passage cells express CD73 and CD90 but not CD45 and CD34 (Figure 2B–E), indicating the characteristic of BMSCs.^31^

**FIGURE 3:**
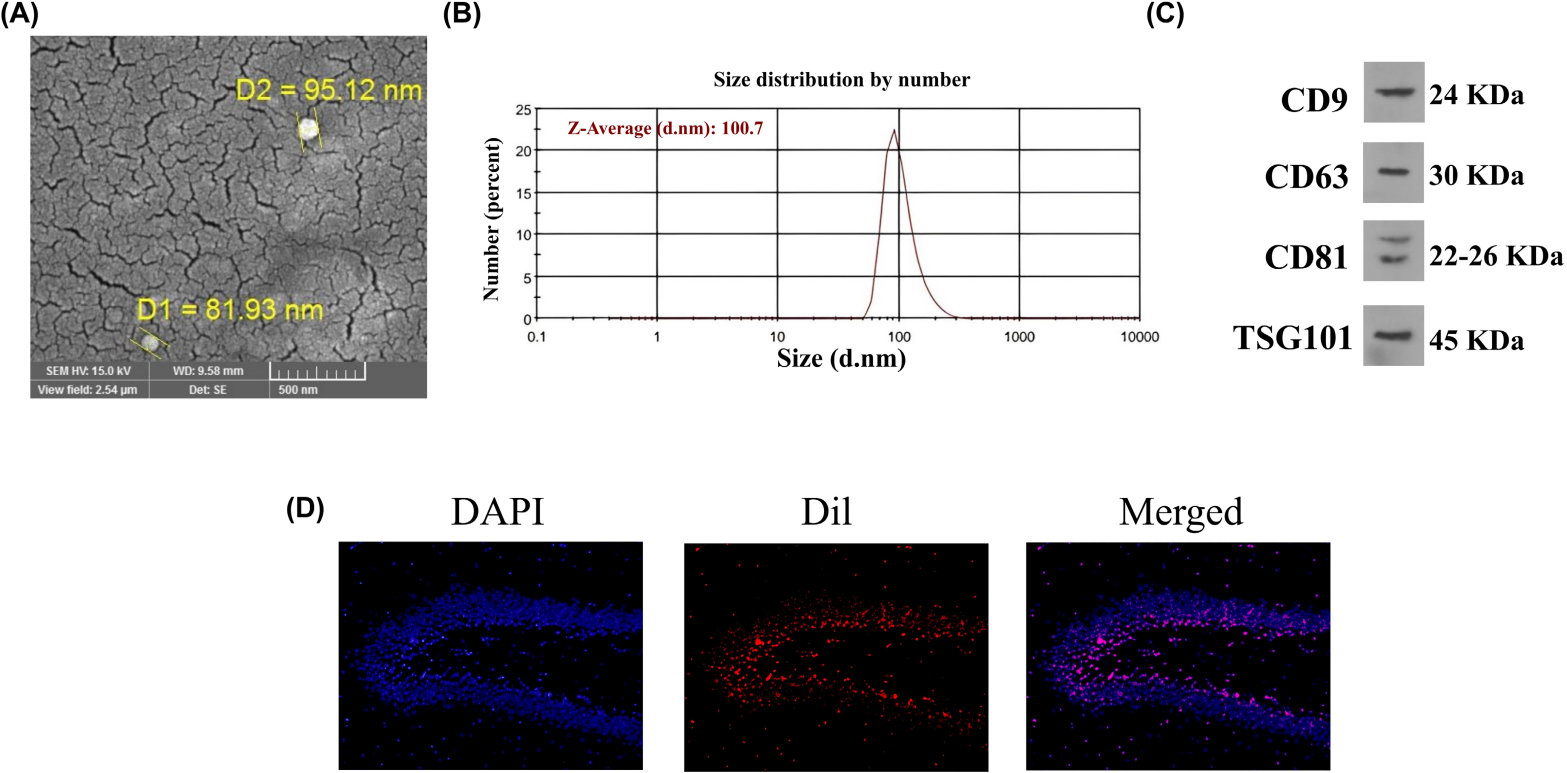
Identification of exosomes. (A) Scanning electron microscopy (SEM) image of isolated exosomes that show morphology and size of the exosome. (B) Size of exosomes after isolation, identified by DLS. (C) Expression of exosome‐specific proteins (CD‐9, CD63, CD‐81, and TSG110) was identified by western blotting. (D) Identification of Dil‐labeled exosomes in the hippocampus.

The results of SEM also showed the existence of exosomes, sized from 81 to 95 nm (Figure 3A). The particle size detection by DLS indicated that particle size ranges from 50 to 160, with a mean size of 100.7 nm (Figure 3B). Additionally, the isolated BMSC‐exosomes exhibited high levels of TSG101, CD81, CD9, and CD63 expression levels (Figure 3C). Besides, the uptake of Dil‐labeled exosomes was identified in the hippocampus (Figure 3D) with the fluorescence microscope.

### 3.2 BMSC‐derived exosomes improved spatial learning and memory in chronic METH‐treated mice

**FIGURE 4:**
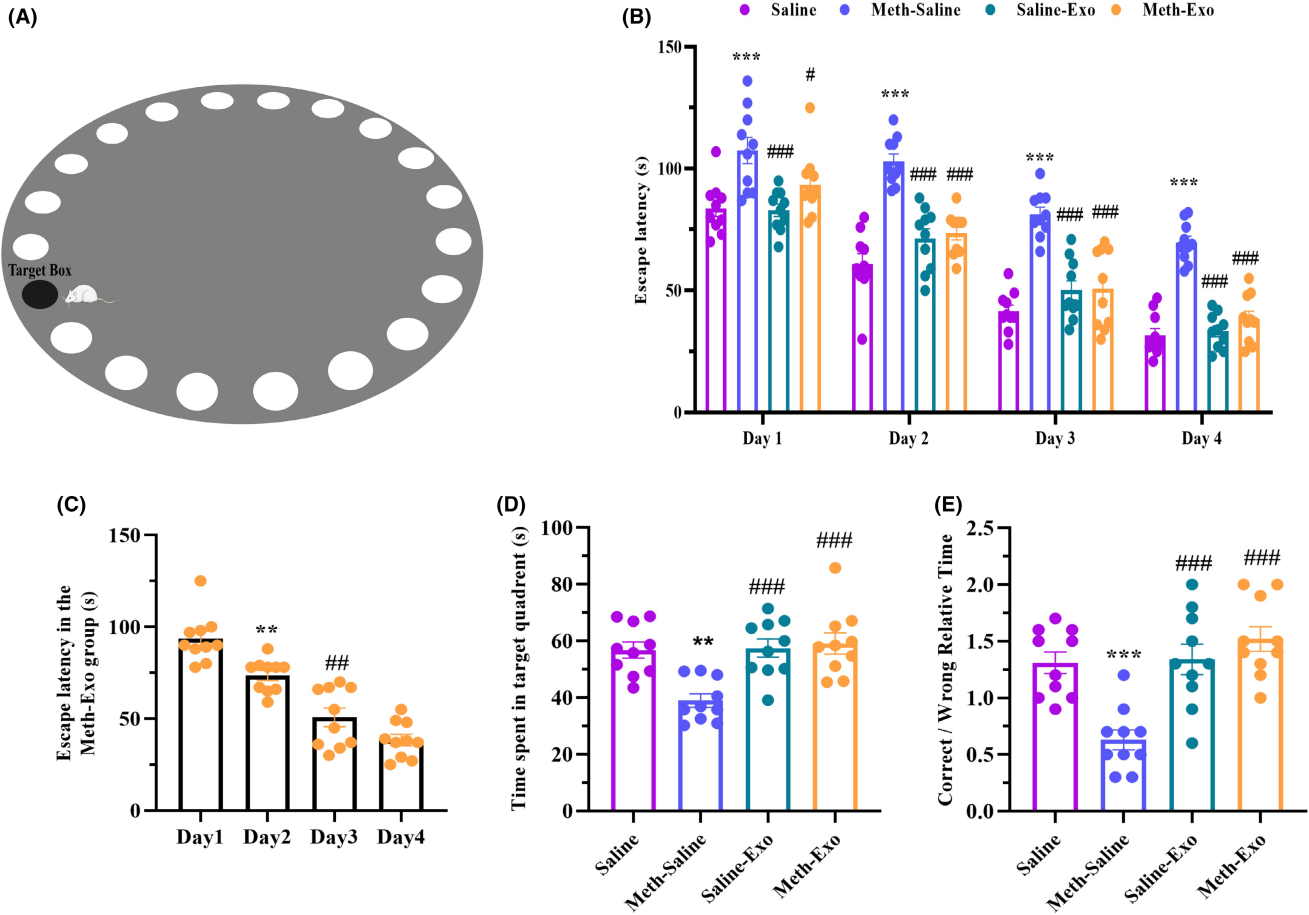
Effect of BMSC‐derived exosomes on spatial memory in chronic methamphetamine (METH)‐treated mice. (A) The Barnes maze apparatus. (B) The escape latency in the acquisition phase (***p < 0.001 vs. Saline group, ^#^ *p* < 0.05 and ^###^ *p* < 0.001 vs. Meth‐Saline group). (C) Mean escape latency alterations of the Meth‐Exo group over four days of training (**p < 0.01 day 1 vs. day 2, ^##^ *p* < 0.01 day 2 vs. day 3). (D) Time spent in the target quadrant in the probe phase (**p < 0.01 vs. Saline group and ^###^ *p* < 0.001 vs. Meth‐Saline group). (E) Correct to wrong relative time in the probe test (***p < 0.001 vs. Saline group, ^###^ *p* < 0.001 vs. Meth‐Saline group). Data are represented as the mean ± SEM (*n* = 10).

Based on the two‐way ANOVA repeated measures of the escape latency (Figure 4), there was a significant effect of the day (*F*_(3, 144)_ = 149.4, *p* < 0.0001), group (F_(3, 144)_ = 81.47, *p* < 0.0001), and no significant effect of days × groups interaction (*F*_(9, 144)_ = 1.429, *p* = 0.1810). Post hoc analysis revealed that escape latency in the Meth‐Saline group was significantly higher than in the Saline and the Saline‐Exo groups in all 4 days of training (*p* < 0.001, Figure 4B). However, compared to the Meth‐Saline group, exosome therapy reduced the escape latency in the METH‐treated group on day 1 (*p* = 0.0303), day 2 (*p* < 0.001), day 3 (*p* < 0.001), and day 4 (*p* < 0.0001). A one‐way ANOVA (*F*_(3, 36)_ = 39.52, *p* < 0.001) also confirmed that the escape latency of the Meth‐Exo group significantly improved during 4 days (*p* < 0.0001, Figure 4C).

Based on the one‐way ANOVA analysis, in the probe test, there was a significant difference among the groups in the time spent in the target quadrant (*F*_(3, 36)_ = 9.455, *p* < 0.0001). Intergroup comparisons verified that the Meth‐Saline group spent a shorter time in the target quadrant than the Saline group (*p* < 0.01, Figure 4D). However, exosome treatment markedly increased the time spent in the target quadrant in the Meth‐Exo (*p* < 0.001) and the Saline‐Exo (*p* < 0.001) groups compared to the Meth‐Saline group.

Also, we found a significant difference in correct to wrong relative time among the experimental groups (*F*_(3, 36)_ = 13.21, *p* < 0.0001). Multiple comparisons showed that the Meth‐Saline group had a lower correct/wrong ratio compared to the Saline group (*p* < 0.001, Figure 4E). Nevertheless, intravenous exosome delivery markedly improved this ratio in the Saline‐Exo (*p* < 0.001) and the Meth‐Exo (*p* < 0.001) groups compared to the Meth‐Saline group.

### 3.3 BMSC‐derived exosomes improved recognition memory in chronic METH‐treated mice

**FIGURE 5:**
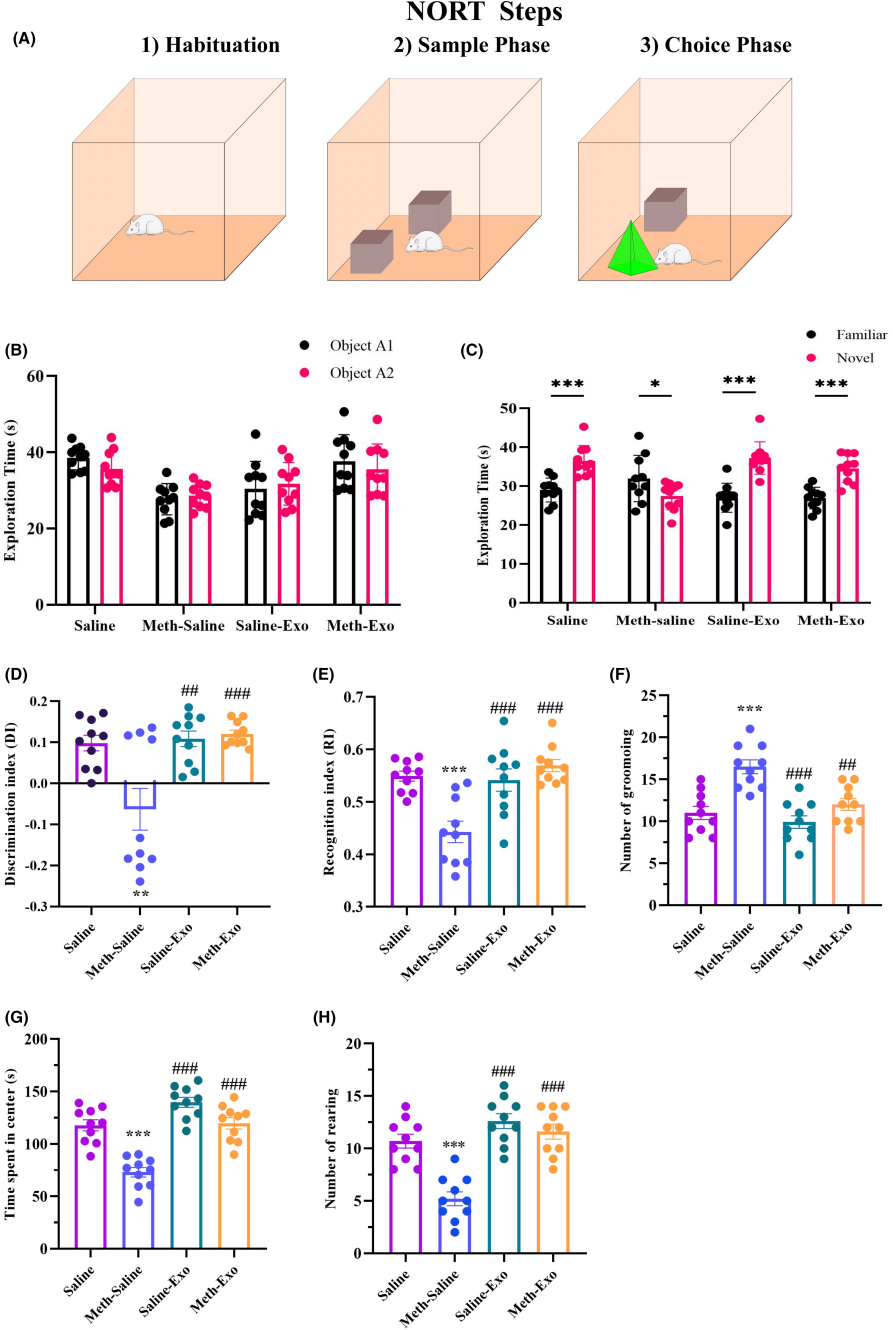
Effect of BMSC‐derived exosomes on recognition memory and anxiety‐like behaviors in methamphetamine‐treated mice. (A) Three phases of the NORT. (B) The exploration time of F1 and F2 objects in the sample phase. (C) Exploration time of the familiar or novel objects in the choice phase (*p < 0.05, ***p < 0.001). (D) Discrimination index (DI) = [(novel − familiar)/(novel + familiar)] (**p < 0.01 vs. Saline group; ^##^ *p* < 0.01 and ^###^ *p* < 0.001 vs. Meth‐Saline group). (E) Recognition memory index (RI) = [novel/(familiar + novel)] (**p < 0.01 vs. Saline group; ^###^ *p* < 0.001 vs. Meth‐Saline group). (F) Number of grooming, (G) number of rearing, and (H) center time (***p < 0.001 vs. Saline group, ^##^ *p* < 0.01 and ^###^ *p* < 0.001 vs. Meth‐Saline group). Data are presented as the mean ± SEM (*n* = 10).

The results of the independent *t*‐test indicated no significant difference in the exploration time of objects A1 and A2 during the sample phase in the experimental groups (*t* = 0.2142, df = 6, Figure 5B), representing no preference for objects A1 and A2. In the choice phase, the Saline (*p* = 0.0003), Saline‐Exo (*p* < 0.0001), and Meth‐Exo (*p* = 0.0002) groups spent more time exploring the novel object compared to the familiar object. However, the exploration time of the familiar object in the Meth‐Saline group was significantly higher than that of the novel object (*p* = 0.0495, Figure 5C). In the one‐way ANOVA, we found significant differences in DI (*F*_(3, 36)_ = 8.826, *p* = 0.0002) and RI (*F*_(3, 36)_ = 11.72, *p* < 0.001) among the groups. Post hoc analysis confirmed that the DI (*p* < 0.01, Figure 5D) and RI (*p* < 0.001, Figure 5E) in the Meth‐Saline group were significantly lower than in the Saline group. However, the DI in the Meth‐Exo (*p* < 0.001) and the Saline‐Exo (*p* < 0.01) was significantly higher than in the Meth‐Saline group (Figure 5D). Likewise, the RI significantly (*p* < 0.001) decreased in the Meth‐Saline group compared to the Saline group. On the other hand, exosome therapy improved RI in the Meth‐Exo group and the Saline‐Exo group (*p* < 0.001 for both comparisons) compared to the Meth‐Saline group.

### 3.4 BMSC‐derived exosomes decreased anxiety‐like behaviors in chronic METH‐treated mice

The habituation phase of the NORT was evaluated to assess anxiety‐like behaviors in the study groups. The results of one‐way ANOVA showed significant differences in the number of grooming (*F*_(3, 36)_ = 14.42, *p* < 0.0001), number of rearing (*F*_(3, 36)_ = 23.67, *p* < 0.0001), and time spent in the center (*F*_(3, 36)_ = 31.28, *p* < 0.0001) across the groups. Post hoc analysis confirmed that chronic METH administration markedly increased the number of grooming (*p* < 0.001, Figure 5F), while decreased the number of rearing (*p* < 0.001, Figure 5G) and time spent in the center (*p* < 0.001, Figure 5H) compared to the Saline group. Nonetheless, exosome therapy significantly increased the number of rearing (*p* < 0.001) and time spent in the center (*p* < 0.001) and decreased grooming frequency (*p* < 0.01) in the Meth‐Exo group.

### 3.5 BMSC‐derived exosomes improved hippocampal neurogenesis‐related markers in chronic METH‐treated mice

**FIGURE 6:**
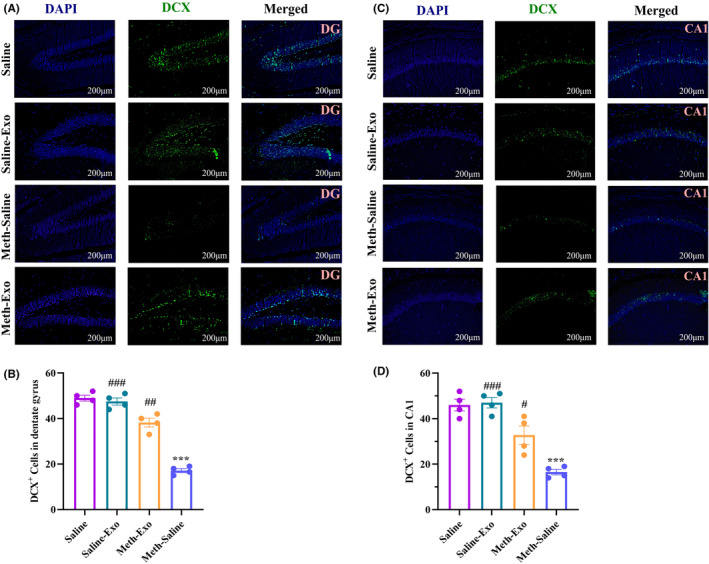
Effect of BMSC‐derived exosomes on DCX expression in the dentate gyrus (DG) and CA1 regions in chronic methamphetamine‐treated mice. Immunofluorescence images of DCX^+^ cells in the DG (A) and CA1 (C) regions. Analysis of the number of DCX‐positive cells in the DG (B) and CA1 (D) regions of different groups. Data are shown as the mean ± SEM (*n* = 4). ***p < 0.001 versus Saline group. ^#^ *p* < 0.05, ^##^ *p* < 0.01, ^###^ *p* < 0.001 versus Meth‐Saline group.

**FIGURE 7:**
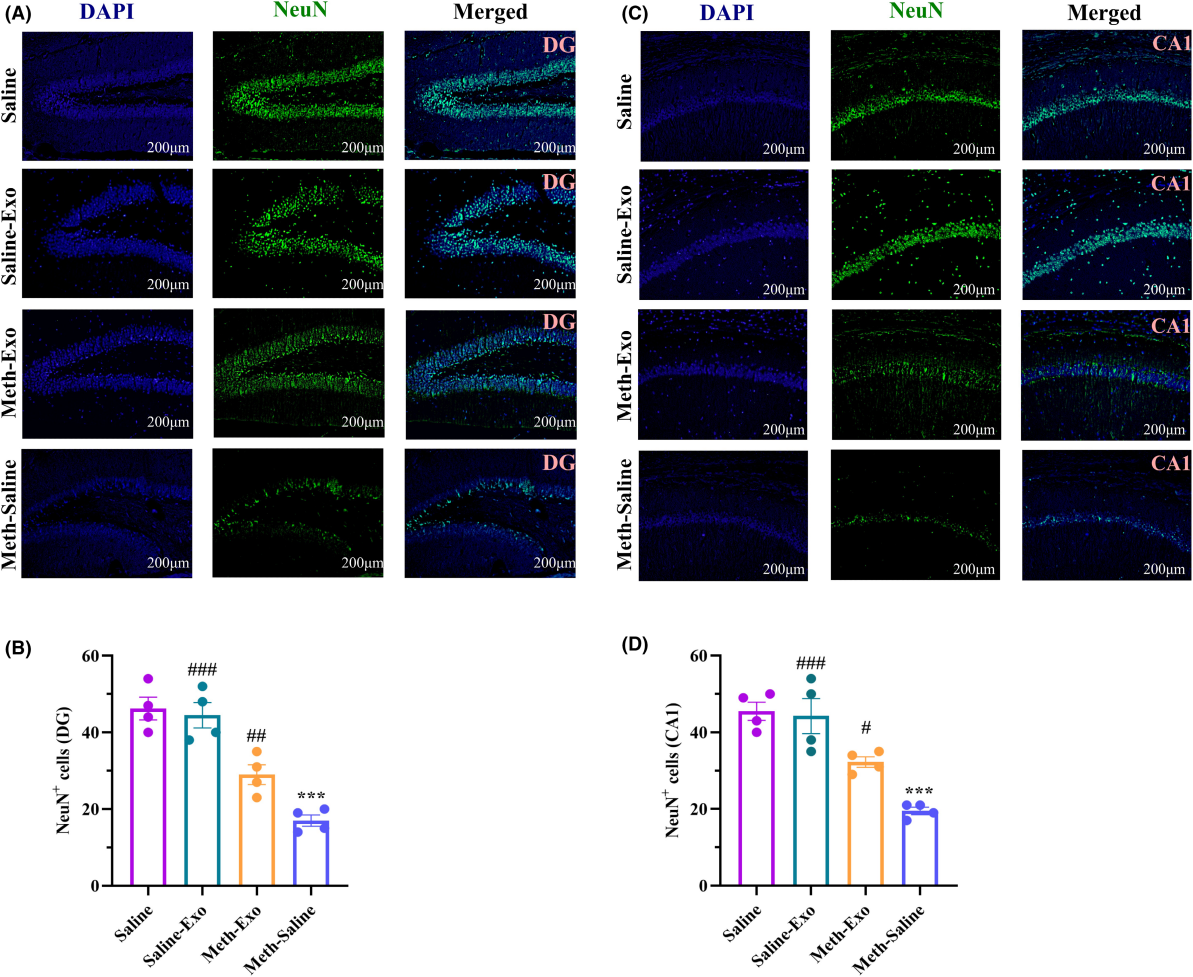
Effect of BMSC‐derived exosomes on NeuN expression in the dentate gyrus (DG) and CA1 regions in chronic methamphetamine‐treated mice. Immunofluorescence images of NeuN‐expressing cells in the DG (A) and CA1 (C) regions. Analysis of the number of NeuN‐positive cells in the DG (B) and CA1 (D) regions of different groups. Data are shown as the mean ± SEM (*n* = 4). ***p < 0.001 versus Saline group. ^#^ *p* < 0.05, ^##^ *p* < 0.01, ^###^ *p* < 0.001 versus Meth‐Saline group.

To study the possible effect of exosome therapy on hippocampal neurogenesis, the expression levels of DCX (expressed by neuronal precursors) and NeuN (a nuclear marker expressed by mature neurons) were assessed by immunofluorescence staining. The one‐way ANOVA of the number of DCX^+^ cells and NeuN^+^ cells in the hippocampal DG [DCX: (*F*_(3, 12)_ = 100.6, *p* < 0.001); NeuN: (*F*_(3, 12)_ = 26.88, *p* < 0.001)] and CA1 [DCX: (*F*_(3, 12)_ = 27.64, *p* < 0.001); NeuN: (*F*_(3, 12)_ = 19.94, *p* < 0.001)] regions showed significant differences among the experimental groups. Post hoc analysis confirmed that METH administration for 30 days markedly decreased the number of DCX^+^ cells (DG: Figure 6A,B, CA1: Figure 6C,D) and NeuN^+^ cells (DG: Figure 7A,B, CA1: Figure 7C,D) in the DG (*p* < 0.001 for both markers) and CA1 (*p* < 0.001 for both markers) regions compared to the Saline group. However, following exosome therapy, the number of DCX^+^ cells and NeuN^+^ cells significantly increased in the DG and CA1 regions of the hippocampus in the Saline‐Exo (*p* < 0.001 for both markers in both the DG and CA1 subfield) and the Meth‐Exo (DCX: *p* < 0.001 for the DG and *p* < 0.01 for CA1 area; NeuN: *p* < 0.05 for both regions) groups compared to the Meth‐Saline group.

### 3.6 BMSC‐derived exosomes decreased neuroinflammation in the hippocampus of METH‐addicted mice

**FIGURE 8:**
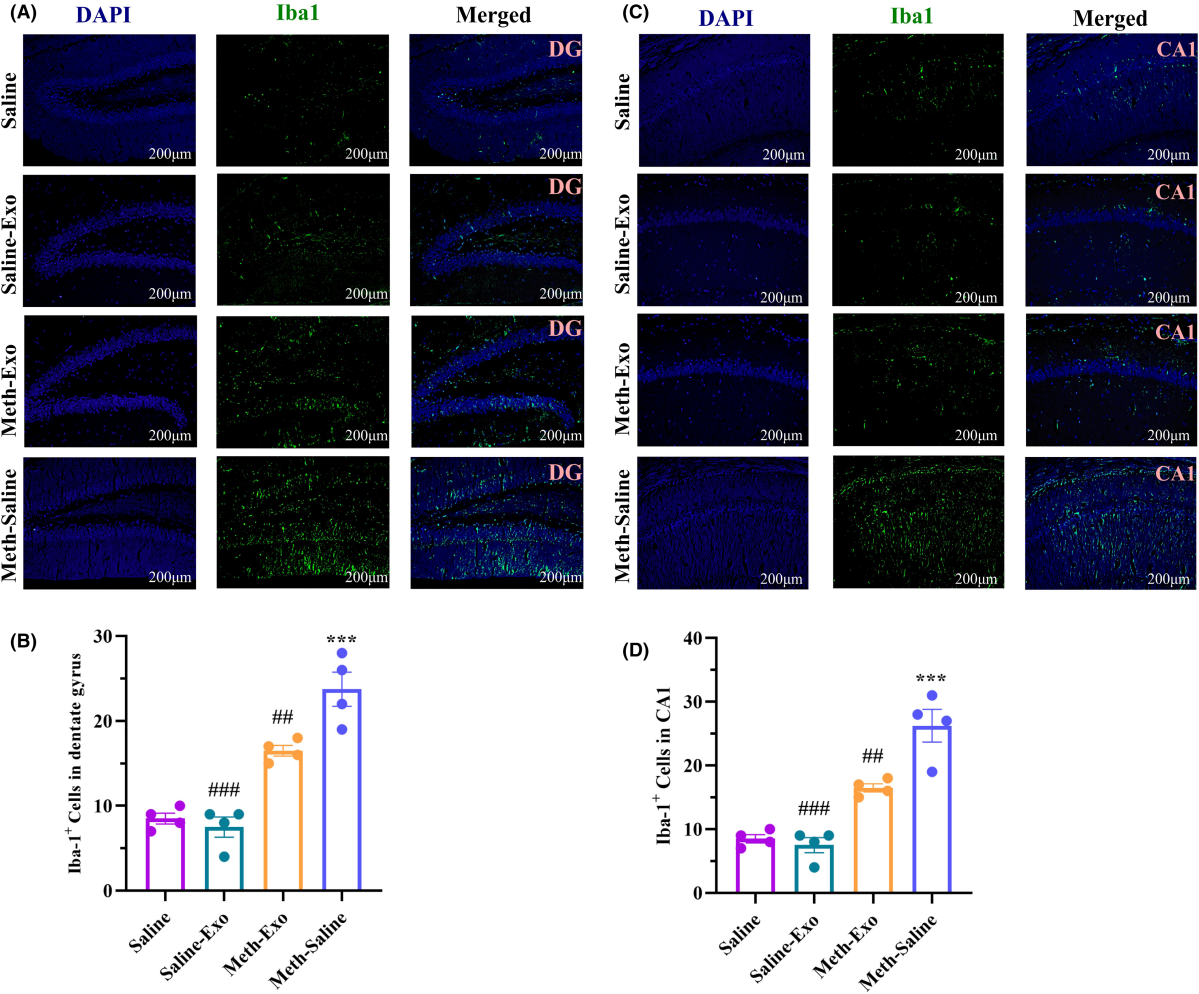
Effect of BMSC‐derived exosomes on Iba‐1 expression in the hippocampus following chronic methamphetamine administration. Immunofluorescence images representing Iba‐1 expression in the DG (A) and CA1 (C) regions. Analysis of the number of Iba‐1^+^ cells in the DG (B) and CA1 (D) regions of the experimental groups. Data are shown as the mean ± SEM (*n* = 4). ***p < 0.001 versus Saline group. ^##^ *p* < 0.01, ^###^ *p* < 0.001 versus Meth‐Saline group.

To examine the effect of exosome therapy on microglial activity in the hippocampus of METH‐addicted mice, Iba‐1 was assessed by immunofluorescence staining in the hippocampal DG and CA1 regions. Our results demonstrated significant differences in Iba‐1 expression in the DG (*F*_(3, 12)_ = 36.71, *p* < 0.001) and CA1 (*F*_(3, 12)_ = 34.33, *p* < 0.001) regions among the groups. Further comparisons between groups revealed increased levels of Iba‐1 in the hippocampal DG (*p* < 0.001, Figure 8A,B) and CA1 (*p* < 0.001, Figure 8C,D) regions of the Meth‐Saline group compared to the Saline‐treated mice. On the other hand, exosome treatment markedly suppressed microglial activity in the METH‐treated mice, as indicated by the reduced number of Iba‐1+ cells in the DG (*p* < 0.01) and CA1 (*p* < 0.01) regions in the Meth‐Exo group compared to the Meth‐Saline group.

**FIGURE 9:**
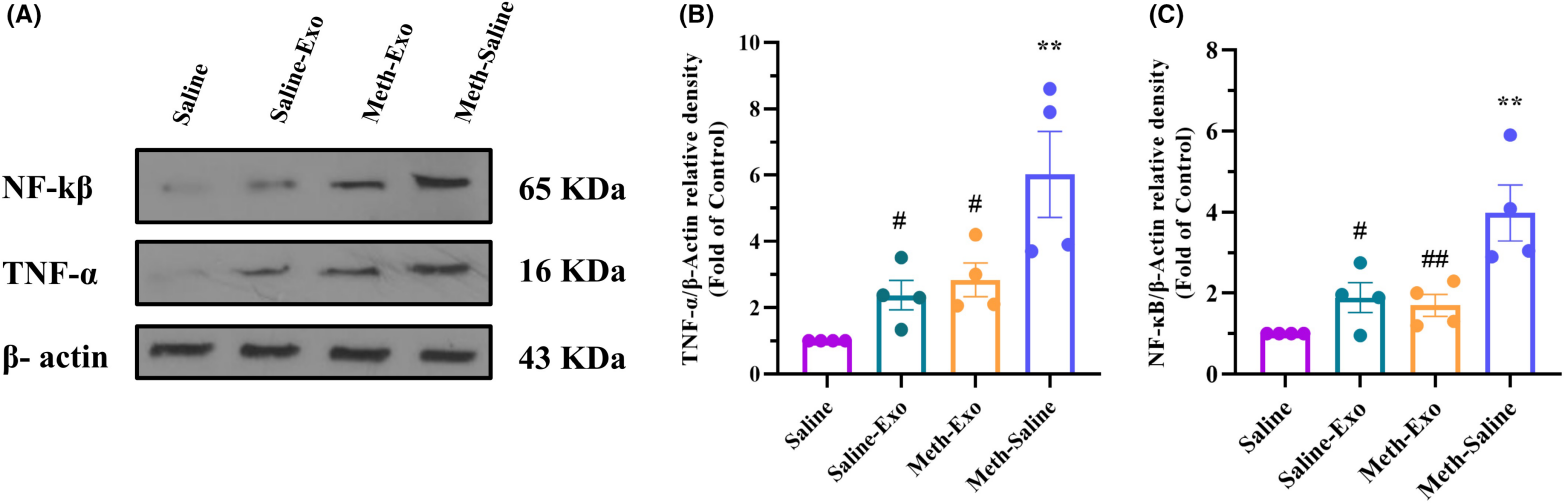
Effect of BMSC‐derived exosomes on protein expressions of NF‐κB and TNF‐α in the hippocampus of methamphetamine‐addicted mice. (A) Immunoblotting images of NF‐κB and TNF‐α expression in the hippocampus. Quantitative densitometric analysis of (B) NF‐κB and (C) TNF‐α (*n* = 4). **p < 0.01 versus Saline group. ^#^ *p* < 0.05 and ^##^ *p* < 0.01 versus Meth‐saline group.

Moreover, protein expressions of TNF‐α and NF‐κB were assessed using western blotting (Figure 9A) in the hippocampal tissue. According to the one‐way ANOVA, protein expressions of TNF‐α (*F*_(3, 12)_ = 8.509, *p* < 0.01) and NF‐κB (*F*_(3, 12)_ = 9.623, *p* < 0.01) were significantly different among the study group. Inter‐group comparisons revealed that chronic METH increased protein levels of NF‐κB (*p* < 0.01, Figure 9B) and TNF‐α (*p* < 0.01, Figure 9C) in the hippocampus. However, intravenous exosome treatment notably down‐regulated TNF‐α and NF‐κB protein levels in the Meth‐Exo (*p* < 0.01 for NF‐κB and *p* < 0.05 for TNF‐α) and the Saline‐Exo (*p* < 0.05 for both proteins) groups as compared to the Meth‐Saline group.

## 4 DISCUSSION

The present investigation demonstrated the beneficial effects of intravenous delivery of BMSC‐derived exosomes as a novel therapeutic strategy for treating METH addiction. This approach significantly enhanced cognitive function and increased the number of newly born neurons expressing DCX and mature neurons expressing NeuN in the hippocampus. Moreover, it caused a reduction in the expression of pro‐inflammatory cytokines such as TNF‐α and NF‐KB and a decrease in microglial activity.

MSCs offer a promising cell‐based therapeutic strategy for brain diseases due to their convenient isolation and lack of immunological rejections.^35^, ^36^ The functional recovery induced by MSCs is mainly mediated by the paracrine activity of the secreted chemicals. Many beneficial effects of MSCs on regeneration are believed to be mediated by exosomes.^37^ Several studies have proven that administering exosomes originating from MSCs effectively enhances the restoration of brain function after injury by enhancing angiogenesis, promoting neuroplasticity, and reducing inflammation in the brain.^38^, ^39^ In neurological diseases, there is a noticeable presence of increased microglial activity and death of brain cells, contributing to a functional deficit.^40^ In this study, we showed that chronic METH administration increased the expression of Iba‐1, as an indicator of microglial activation, in the CA1 and DG regions of the hippocampus. However, this effect was counteracted by exosome therapy.

Apart from Iba1, an increase in TNF‐α expression in various brain regions confirms the stimulation of microglia and neuroinflammation.^41^ According to studies, TNF‐α activity promotes the release of inflammatory cytokines like IL‐6 and IL‐1β and upregulates the NF‐κB signaling pathway, which in turn helps to activate microglia.^42^, ^43^ Evidence also indicates that METH induces a significant neuroinflammatory reaction, often characterized by gliosis and increased cytokine levels. An in vitro study discovered that METH stimulates the activator protein‐1 and NF‐kB pathway, leading to increased expression of TNF‐α in human brain endothelial cells.^44^ METH stimulates the secretion of TNF‐α by both brain endothelial cells and astrocytes, leading to the activation of the NF‐κB pathway. As a result, the transport of substances across both the transcellular and paracellular pathways in endothelial cells is enhanced, causing an increased permeability of the BBB. However, the disruption of the BBB caused by METH can be effectively prevented by inhibiting TNF‐α or NF‐κB.^45^

On the other hand, previous investigations have demonstrated that both MSCs and exosomes produced from MSCs can enhance the production of anti‐inflammatory molecules, such as IL‐10 and TGF‐β, while suppressing the expression of pro‐inflammatory cytokines, such as IL‐6 and TNF‐α.^46^ The current study, confirming similar findings, verified that chronic METH administration significantly increases hippocampal expression of TNF‐α and NF‐κB, while BMSC‐derived exosomes reduced the expression of these factors in the hippocampal tissue of METH‐treated mice.

A variety of hippocampus‐dependent processes, such as learning, memory, and mood regulation, depend on neurogenesis, which mostly happens in the SVZ of the lateral ventricles and the subgranular zone of the hippocampal DG in the adult brain.^47^, ^48^, ^49^ Increased inflammation and concurrent decrease in neurogenesis are prominent features of numerous neurodegenerative disorders.^50^ Previous studies demonstrated that the activation of microglia and inflammation hinder the growth of new neurons in the hippocampus by reducing the survival of immature neurons.^49^, ^51^, ^52^ Moreover, the administration of lipopolysaccharide was reported to decrease the number of DCX^+^ cells and increase the number of microglia in the DG. However, this effect can be reversed by the use of anti‐inflammatory medications.^51^ Furthermore, many pro‐inflammatory cytokines, including TNF‐α, IL‐1β, and IL‐6, have a detrimental effect on the survival, growth, and development of neural stem cells.^53^, ^54^ Likewise, chronic METH has been reported to decrease neurogenesis in the hippocampus,^55^ mainly by decreasing the number of Ki‐67^+^ cells and lessening the number of DCX^+^ immature neurons.^56^ Singhakumar et al. discovered that administering melatonin pretreatment effectively reversed the cognitive decline caused by METH. This was achieved by enhancing neurogenesis in both the SVZ and DG, boosting the function of post‐synaptic proteins, and preventing astrogliogenesis in the brain.^57^ In neurological disorders such as Alzheimer's disease, MSC‐derived exosomes can enhance the expression of DCX and PSA‐NCAM, thereby proving their regenerative potential.^58^ In the current study, we also found that METH decreased DCX and NeuN expression levels in the DG and CA1 regions of the hippocampus. Nevertheless, MSC‐derived exosomes increased the number of DCX^+^ and NeuN^+^ cells in the hippocampal regions, indicating an increase in neurogenesis, potentially via reducing pro‐inflammatory cytokines and blocking microglia activation.

METH addiction not only increases neuroinflammation and disrupts neurogenesis, but it also inhibits neuronal plasticity and remodels specific brain circuits.^59^, ^60^ Accumulating evidence indicates that addicted individuals exhibit impaired different cognitive domains, including social cognition, executive function, attention, cognitive flexibility, and working memory.^61^ One of the primary objectives of various METH addiction treatment modalities is to enhance cognitive function.^14^, ^62^ In this study, we showed that BMSC‐derived exosome therapy recovered cognitive dysfunction in the METH‐treated mice. An important benefit of exosome therapy, stemming from its rich cargo, is the enhancement of cognitive function, as well as its ability to reduce inflammation and promote neuroregeneration.^21^, ^63^ We also indicated that intravenous exosome effectively improved the cognitive function of the METH‐addicted mice in the Barnes maze and the NORT.

## 5 CONCLUSION

**FIGURE 10:**
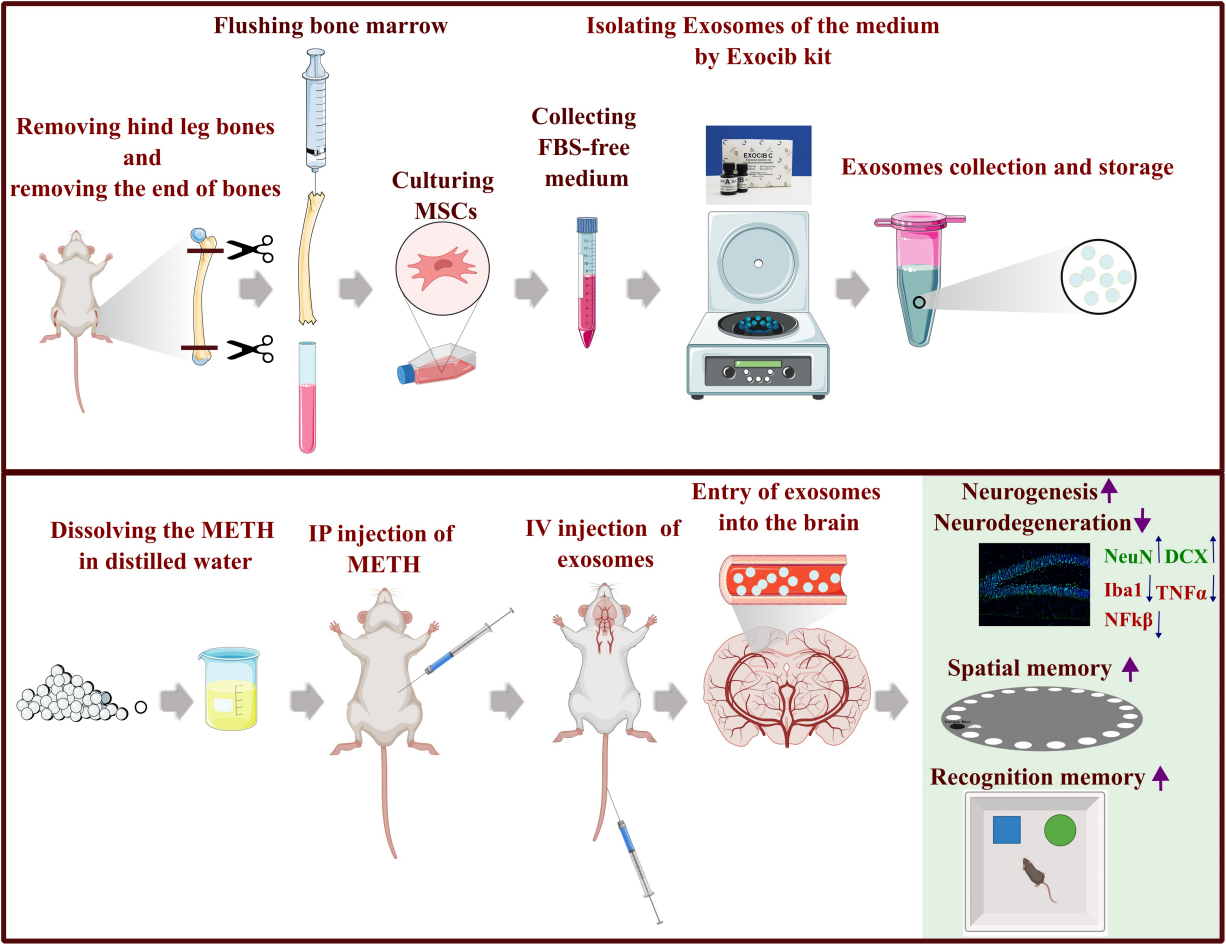
A schematic representation of mesenchymal stem cells isolation from mice bone marrow and culture of these cells. After culturing, exosomes were extracted from a free‐FBS medium and injected via the tail vein in mice that were chronically addicted to methamphetamine. These exosomes improved neurogenesis in the hippocampal tissue by increasing the expression of DCX and NeuN. Also, they reduced inflammation by decreasing the expression level of Iba1, TNFα, and NFkβ. These alterations, following exosome therapy, helped to improve the spatial and recognition memory of addicted animals.

The results of our study confirm that exosomes derived from BMSCs provide a neuroprotective effect in METH‐treated mice by suppressing neuroinflammation and enhancing neurogenesis in the hippocampus. Hence, BMSCs‐exosomes might serve as a promising approach for the treatment of METH neurotoxicity by lessening neuroinflammatory factors (TNF‐α, NF‐κB) and microglial polarization (Iba‐1) and also augmenting neurogenesis (DCX^+^, NeuN^+^) and cognitive function (Figure 10).

## AUTHOR CONTRIBUTIONS

G.M., F.G., H.S.Z., and S.F. designed research and wrote the manuscript. S.F. and H.S.Z. performed the research and analyzed the data. F.F. and F.G. edited the manuscript.

## FUNDING INFORMATION

This study was supported by a grant (67159) from the Drug Applied Research Center, Tabriz University of Medical Sciences (Tabriz, Iran), and a grant from the Council for Development of Stem Cell Sciences Technologies.

## CONFLICT OF INTEREST STATEMENT

The authors declare no competing interests.

## Supporting information


**Data S1.**.

## Data Availability

The datasets used and/or analyzed during the current study are available from the corresponding author on reasonable request.
