# Correction to Mesenchymal stem cell‐derived exosomes improve neurogenesis and cognitive function of mice with methamphetamine addiction: A novel treatment approach. *CNS Neurosci Ther*. 2024 May;30(5):e14719. doi: 10.1111/cns.14719

**DOI:** 10.1111/cns.14845

**Published:** 2024-07-03

The authors affiliation list was updated.

In the author list, the affiliation address of Reza Rahbarghazi was changed to two institutional affiliations

Solmaz Fallahi^1,2^, Hamid Soltani Zangbar^3^, Fereshteh Farajdokht^2,4^, Reza Rahbarghazi^5,6^, Fariba Ghiasi^1,2^, Gisou Mohaddes^1,2,3,7^

^1^Drug Applied Research Center, Tabriz University of Medical Sciences, Tabriz, Iran

^2^Department of Physiology, Faculty of Medicine, Tabriz University of Medical Sciences, Tabriz, Iran

^3^Department of Neuroscience and Cognition, Faculty of Advanced Medical Sciences, Tabriz University of Medical Sciences, Tabriz, Iran

^4^Neurosciences Research Center, Tabriz University of Medical Sciences, Tabriz, Iran

^5^Stem Cell Research Center, Tabriz University of Medical Sciences, Tabriz, Iran

^6^Department of Applied Cell Sciences, Faculty of Advanced Medical Sciences, Tabriz University of Medical Sciences, Tabriz, Iran

^7^Department of Biomedical Education, California Health Sciences University, College of Osteopathic Medicine, Clovis, California, USA

We apologize for this error.

