# Supplementary figures and images for: Mesenchymal stem cell‐derived exosomes improve neurogenesis and cognitive function of mice with methamphetamine addiction: A novel treatment approach
Source: CNS Neurosci Ther. 2024 May 23;30(5):e14719. doi: 10.1111/cns.14719 (PMC11116483; doi:10.1111/cns.14719)

Full unedited gels for Fig.9 A

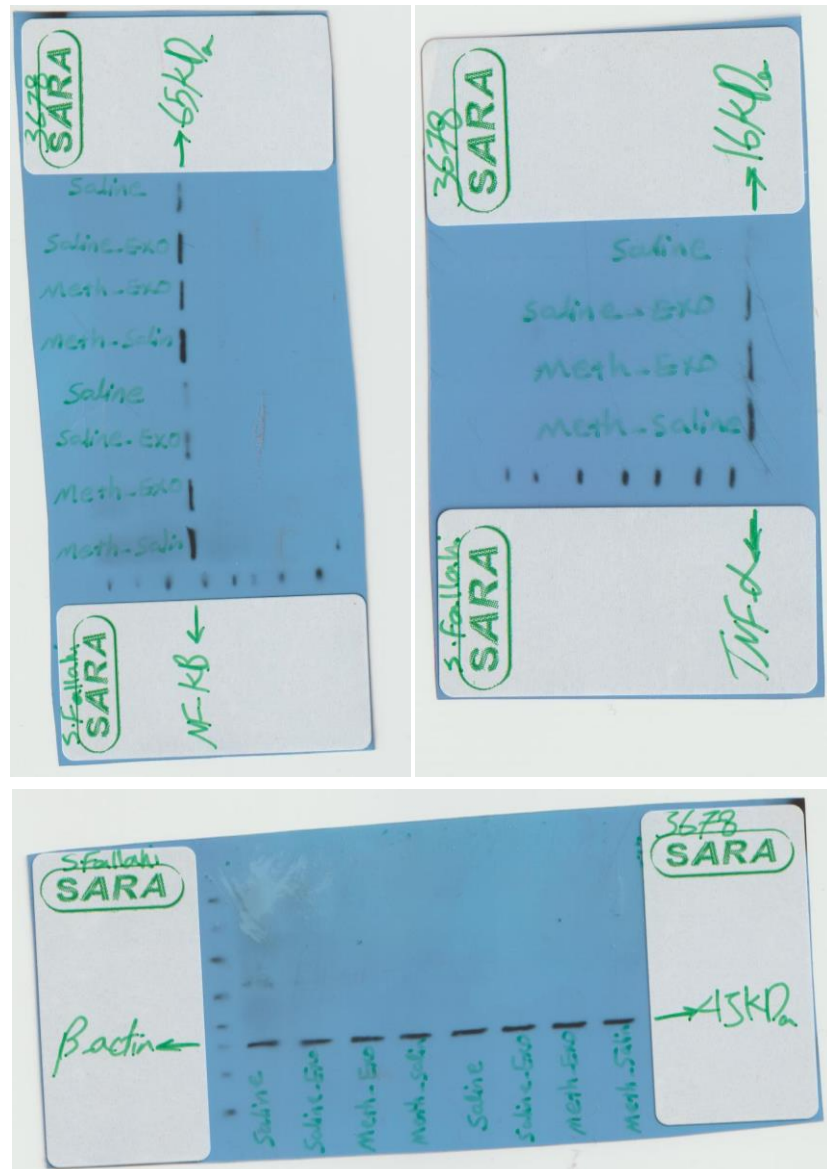

Full unedited gels for Fig.3 C

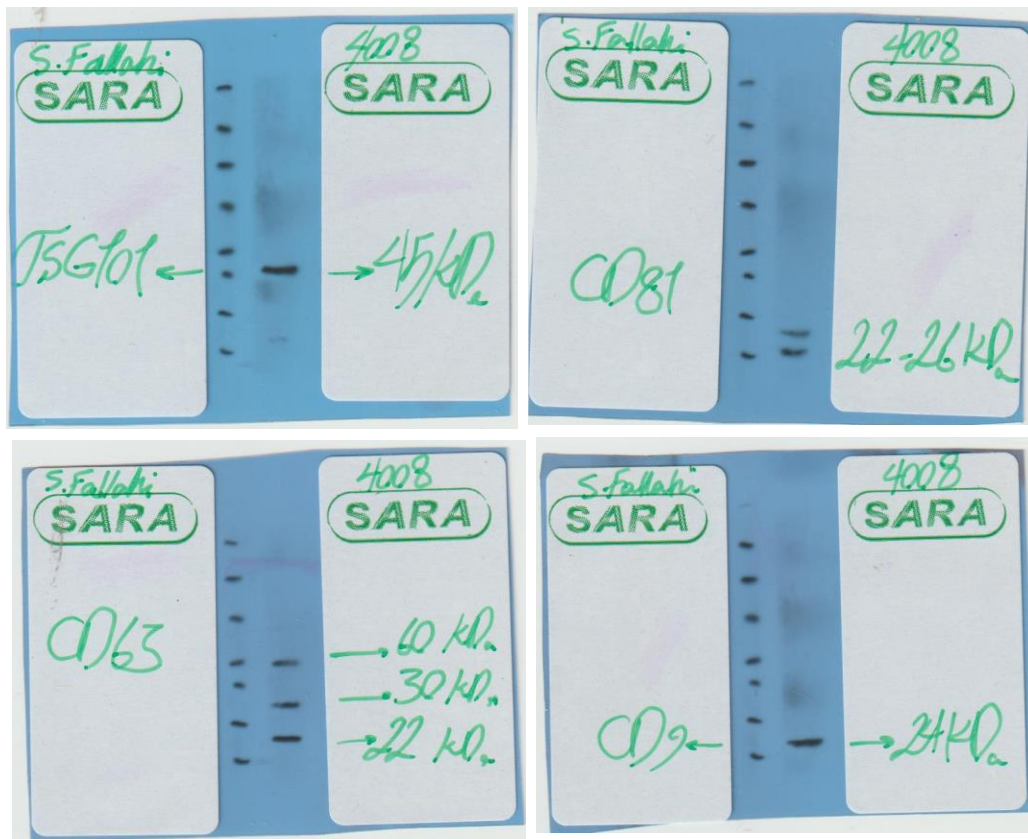

Supplement: Supplementary file 1 — Data S1.. [file CNS-30-e14719-s001.pdf]
